# The Impact of Chronic Heat Stress on the Growth, Survival, Feeding, and Differential Gene Expression in the Sea Urchin *Strongylocentrotus intermedius*

**DOI:** 10.3389/fgene.2019.00301

**Published:** 2019-04-04

**Authors:** Yaoyao Zhan, Jiaxiang Li, Jingxian Sun, Weijie Zhang, Yingying Li, Donyao Cui, Wanbin Hu, Yaqing Chang

**Affiliations:** Key Laboratory of Mariculture & Stock Enhancement in North China’s Sea, Ministry of Agriculture and Rural Affairs, Dalian Ocean University, Dalian, China

**Keywords:** heat stress, *Strongylocentrotus intermedius*, growth, survival, feeding, transcriptome

## Abstract

To explore the impact of chronic heat stress on commercial echinoderms, the present study assessed the effects of chronic high temperature on the growth, survival, feeding, and differential gene expression in the sea urchin *Strongylocentrotus intermedius* cultured in northern Yellow Sea in China. One suitable seawater condition (20°C) and one laboratory-controlled high temperature condition (25°C) were set up. After 28 days incubation, our results showed that: (1) The specific growth, survival, and ingestion rates of *S. intermedius* reared under high temperature (25°C) decreased compared to those reared under optimal temperature (20°C) conditions; (2) comparative transcriptome analysis identified 2,125 differentially expressed genes (DEGs) in *S. intermedius* reared under high temperature (25°C) compared to those subjected to optimal temperature condition (20°C), which included 1,015 upregulated and 1,100 downregulated genes. The accuracy of the transcriptome profiles was verified by quantitative real-time PCR (qRT-PCR). Further Gene Ontology (GO) and Kyoto Encyclopedia of Genes and Genomes (KEGG) pathways analyses revealed that these DEGs mainly enriched the functional categories of ribosome, protein processing in endoplasmic reticulum, and prion diseases. A total of 732 temperature-induced expressed genes, such as ATP5, heat shock protein 70, and heat shock protein 90, were identified as candidates that were closely correlated with heat resistance in *S. intermedius*. Differentially expressed transcription factors (TFs), such as AP-1, Fos, CREB, and ZNF, were also identified as potential regulators that regulate the molecular network that was associated with responses to heat stress in sea urchins. Observations in the present study provide additional information that improves our understanding of the molecular mechanism of temperate echinoid species in response to heat stress, as well as theoretical basis for the molecular-assisted breeding of heat-resistant sea urchins.

## Introduction

Seawater temperature has been proven to be a major environmental factor affecting echinoderms from the biomacromolecular to ecological levels. With climate-induced ocean warming, extensive efforts have been made in studying the impact of elevated seawater temperatures on echinoderms. Laboratory-based studies have demonstrated that increased seawater temperature affects early development, survival, growth, metabolism, immunity, behavior, and gene expression profiles in echinoderms. For example, with the elevation of near-future seawater temperature, fertilization and early development in the sea urchin *Heliocidaris erythrogramma* would be compromised (Byrne et al., 2009). Increased seawater temperatures can affect and reduce both the specific growth rate (SGR) and contents of highly unsaturated fatty acids (HUFAs) in juvenile sea cucumber (Yu et al., 2016). It has been demonstrated that the parental effect of long acclimatization could increase thermal tolerance in juvenile sea cucumber *Apostichopus japonicus* (Wang et al., 2015). The existence of species-specific innate immune response variations was investigated in the tropical subtidal sea urchin *Lytechinus variegatus* and the intertidal sea urchin *Echinometra lucunter* while coping with rising sea temperatures (Branco et al., 2013). In addition, negative effects of elevated seawater temperature on covering and righting behaviors were observed in the sea urchins *L. variegatus* and *Strongylocentrotus intermedius* (Brothers and Mcclintock, 2015; Zhang et al., 2017). Comparative transcriptome study indicated alterations in gene expression profiles under mild, chronic increases in temperature stress in embryos of the sea urchin *Strongylocentrotus purpuratus* (Runcie et al., 2012). A recent study also showed that the response of juvenile sea urchin *Loxechinus albus* to acute increases in sea temperature is an integrated differential gene regulatory network that includes heat-shock, membrane potential, and detoxification (Vergara-Amado et al., 2017).

The temperate edible sea urchin *S. intermedius* is naturally distributed along the intertidal and subtidal rocky bottom of Hokkaido, Japan, the Korean Peninsula, and Russian Far East (Chang et al., 2004; Lawrence, 2013). This species has an average lifespan of 8–10 years, and the sexual maturity age is 1.5–2 years. The thermal tolerance of this species is from -1 to 23°C (Chang et al., 2004), and the suitable sea temperature range for the growth of this species is 15–20°C. In 1989, this species was introduced from Japan to north China by the Dalian Ocean University, and artificial breeding was subsequently performed. To date, *S. intermedius* has been the predominant commercial valuable sea urchin species that has been widely cultivated along the coastal areas of the north Yellow Sea in China (Chang et al., 2004). Due to global ocean warming, sea water temperatures in the north Yellow Sea in China have often been higher than 25°C (the lethal limit of *S. intermedius*) in the summer in the past few years (Zeng et al., 2006), resulting in the massive death of cultured *S. intermedius*. The sustainable development of *S. intermedius* farming and industry, therefore, is under serious threat. Our previous study demonstrated the existence of genotype by temperature interactions (GEI) in the survival rate (SR) in the selection of *S. intermedius* (Chang et al., 2016); however, the response to heat-stress, especially the corresponding gene expression mechanism in *S. intermedius* remain unclear.

In the present study, we investigated the impact of high water temperatures on the growth, survival, and feeding of *S. intermedius*. Then, we identified candidate genes that were closely correlated to heat tolerance in *S. intermedius* by comparative transcriptome analysis between suitable (20°C, as control) and high temperature (25°C) seawater conditions in *S. intermedius*. A gene regulatory network related to heat-tolerance was also predicted by setting up relationships between candidate genes and differential expressed transcription factors (TFs). The findings of this study enrich our knowledge of the molecular responses of sea urchins to heat stress, as well as provide candidate genes that can serve as molecular markers that could be potentially used in the selection of heat tolerance-resistant breeding of *S. intermedius*.

## Materials and Methods

### Sea Urchins and Treatments

A total of 360 *S. intermedius* (average test diameter: 10 ± 0.1 mm) were transported from Dalian Haibao Fisheries Company to the Key Laboratory of Mariculture & Stock Enhancement in the North China’s Sea, Ministry of Agriculture and Rural Affairs at the Dalian Ocean University in August 2015. All of the sea urchins were kept in ∼60-L recirculating sea water tanks; each tank was fitted with an automatic temperature control and monitoring system (Dalian Huixin Titanium Equipment Development Co., Ltd., Liaoning, China). Seawater was sand filtered and continuously aerated. The animals were kept under natural light. All of the specimens were fed kelp (*Laminaria japonica*). Sea urchins were acclimated to default laboratory conditions [18 ± 0.5°C and 31.22 ± 0.14 (practical salinity units) PSU] for 1 week prior to experimentation. The experiments were conducted between November 2015 and January 2016.

All of the sea urchins were dried with a paper towel and weighed on a digital balance (0.01 g sensitivity; AL204; Mettler Toledo, Shanghai, China) to obtain initial mass (*W*_1_). We then randomly divided the sea urchins into three groups of 60 specimens each (three replicates for each temperature). Each group was housed in a separate tank. To reach the desired temperature (20 and 25°C), we removed half of the seawater from each tank every day, and replaced it with seawater at a different temperature. We changed the temperature of the new seawater such that the temperature of the entire tank did not increase by more than 1°C per day; this was based on a previous study on *S. intermedius* (Lawrence et al., 2009) and on field survey data of the coastal waters of the Yellow Sea (Zhang et al., 2016).

We monitored the temperature in each tank using an automatic temperature control and with a water quality monitor (A329 Portable Meter; Thermo Scientific Orion Star, Beijing, China).

### Growth, Survival, and Ingestion in Each Treatment

Before the experiment, *S. intermedius* individuals in each treatment were dried with a paper towel and weighed on a digital balance (0.01 g sensitivity; AL204; Mettler Toledo, Shanghai, China) to obtain initial average mass (*W*_0_). The specific growth rate (SGR), survival rate (S), average food consumption (FC) of individual, and daily feeding rate (FR) were calculated using the following formulae:

$$\begin{array}{l} \;\;\;SGR\;(\%\cdot day^{-1})=100\times (\ln\;W_{t}-\ln\;W_{0})/t;\\ \;\;\;\;\;\;S(\%)=100\times (N_{t}/N_{0});\\ \;\;\;\;FC(g\cdot individual^{-1})=(TB_{t}-RB_{t})/N_{t};\\ FR(\%\cdot day^{-1})=100\times \sum (TB_{t}/N_{t}-RB_{t}/N_{t})/[(W_{t}+W_{0})/2\times t]; \end{array}$$

where *W*_t_ is the average body weight (g) of live *S. intermedius* on day *t*; *t* is the duration of experiment; *N*_0_ is the initial number of live *S. intermedius*; *N*_t_ is the number of live *S. intermedius* on day *t*; *TB*_t_ is the bait supplied on days *t*; and *RB*_t_ is the total amount of remaining bait on day *t* (Qin et al., 2011; Chang et al., 2016).

### Sample Collection for RNA-Seq

As for individual test diameter of around 10 mm, most of the sea urchins sampled in this study were too small to develop their gonads. In addition, it is generally difficult to dissect or obtain enough tissues such as tube feet, coelomocytes, and perioral membranes for RNA-seq library construction and subsequent validation.

The intestines are important organs for nutrient intake and stress defense in sea urchins, and these are the only sources for tissues that can be sampled under a dissection microscope in this study. Therefore, we opted to utilize the intestines for perform transcriptome analysis in this study. At the end of the experimental period, the number of living *S. intermedius* individuals in three high temperature group (25°C) replicate tanks was 40 (replicate 1#), 31 (replicate 2#), and 34 (replicate 3#). To ensure that there are three independent samples for the transcriptome validation experiment, we randomly selected 20 *S. intermedius* specimens from each replicate tank for intestinal RNA extraction. For RNA sequencing library construction, the intestines of 20 living *S. intermedius* specimens from replicate 1# of high temperature group (25°C) were carefully removed and pooled (designated as Si_TT2_1), and 20 intestines of living *S. intermedius* specimens from replicate 2# and replicate 3# (10 of each) of high temperature group (25°C) were carefully removed and pooled (designated as Si_TT2_2). Two optimal temperature (20°C) sample pools (as control) were constructed using the procedure employed for high temperature (25°C) sample pool construction. We named two optimal temperature (20°C) sample pools as Si_TT0_1 and Si_TT0_2. All of the pooled samples were stored at -80°C until RNA extraction.

### RNA Extraction and Sequencing

Total RNA was extracted from each pooled sample by using TRIzol (Ambion, United States) following the manufacturer’s instructions. Total RNA quantity and integrity were assessed by 1% agarose gel electrophoresis and the RNA Nano 6000 assay kit of the Agilent Bioanalyzer 2100 system (Agilent Technologies, CA, United States).

A total amount of 2 μg high quality RNA per pooled sample was used for RNA sample preparation. All high-quality RNA samples were sent to BGI Co., Ltd. (Beijing, China). Sequencing libraries were constructed using the NEBNext^®^ Ultra^TM^ RNA Library Prep Kit for Illumina^®^ (NEB, United States) according to the manufacturer’s recommendations, and index codes were added to sequences to distinguish one sample from another. The Agilent Bioanalyzer 2100 system was employed to assess the quality of each RNA library. The index-coded samples were clustered on a cBot Cluster Generation System using TruSeq PE Cluster Kit v3-cBot-HS (Illumina) according to the manufacturer’s instructions. After cluster generation, the library preparations were sequenced on an Illumina Hiseq platform and paired-end reads (Hiseq 4000, 101PE).

### Transcriptome Assembly and Annotation

Clean data (clean reads) were obtained from the raw data (raw reads) by removing adaptors, reads with poly-N, and low-quality reads. All of the clean reads were then submitted to the National Center for Biotechnology Information (NCBI) Short Read Archive (SRA) Sequence Database (Accession Number PRJNA508827). The percentage of bases with a Phred value > 20 (Q20), the percentage of bases with a Phred value > 30 (Q30), and the content of base G and C (GC-content) were calculated. The high-quality clean data were used for subsequent analyses. Clean reads were assembled into transcriptome as reference using Trinity (Grabherr et al., 2011) with min_kmer_cov set to 2 by default, and all of the other parameters set to default. The clean data were mapped back onto the assembled transcriptome, and read count for each gene was obtained from the mapping results by RSEM (v1.2.12). BUSCO v.3.0.2 was used to assess the completeness of the gene assembly (Simão et al., 2015).

Transcriptome annotation was performed using Basic Local Alignment Search Tool (BLAST) searches against the NCBI non-redundant (Nr) databases, NCBI nucleotide sequences (Nt), Swiss-Prot, InterPro, Kyoto Encyclopedia of Genes and Genomes (KEGG), Clusters of Orthologous Groups (COG), and Swiss-Prot. We employed Blast2GO with NR annotation for Gene Ontology (GO) annotation, and InterProScan5 for InterPro annotation.

### Single Nucleotide Polymorphism (SNP) and Simple Sequence Repeat (SSR) Identification

Single nucleotide polymorphism (SNPs) and SSRs in the transcriptome level were identified using GATK3 software (v3.4) (Mckenna et al., 2010) and MISA (microsatellite)^1^, respectively. The parameters for SNP identification were an MQ < 20.0 and QD < 2.0. SSR identification criteria in the MISA script were 1–12, 2–6, 3–5, 4–5, 5–4, and 6–4.

### Differentially Expressed Gene (DEG) Analysis

Gene expression levels were calculated as previously described (Li and Dewey, 2011) with RSEM (v1.2.12). Differential expression analysis between optimal temperature (20°C; Si_TT0) and high temperature (25°C; Si_TT2) was performed using the NOISeq R package (v3.1). NOISeq provides statistical routines for determining differential expression in digital gene expression data using a model based on a noise distribution model (Tarazona et al., 2011). The software information: fold-change ≥ 2.0 and probability ≥ 0.8. Since genes with an adjusted | fold-change|≥ 2.0 and probability ≥ 0.8 found by NOISeq were assigned as differentially expressed.

### GO and KEGG Pathway Enrichment Analyses

Differentially expressed genes were classified based on GO and KEGG functional annotation, GO, and pathway functional enrichment was performed using phyper, as implemented in the R package (v3.1). *P*-values were calculated using the hypergeometric test:

$$\begin{array}{c} P=1-\sum_{i=0}^{m-1}\frac{\left(\begin{array}{c} M\\ i \end{array}\right)\left(\begin{array}{c} N-M\\ n-i \end{array}\right)}{\left(\begin{array}{c} N\\ n \end{array}\right)}. \end{array}$$

To ensure relatively precise results, we calculated the false discovery rate (FDR) for each *p*-value. In general, the terms in which FDR was not larger than 0.001 were defined as significant. Enriched cluster analysis of candidate DEGs was performed using the R package (v3.1).

### qRT-PCR Validation

Annotated DEGs were validated using quantitative real-time reverse transcription polymerase chain reaction (qRT-PCR). Twelve DEG candidates were randomly selected (including eight upregulated and four downregulated DEGs) from 2,125 DEGs. The DEG input RNA was used as template for cDNA synthesis. cDNA was synthesized using PrimeScript^TM^ RT reagent Kit (TaKaRa, Japan). The cytochrome b (*Cytb*) gene was used as internal control (Yang et al., 2010). Primers used in qRT-PCR analyses were designed by Primer Premier 5.0 (Table 1). qRT-PCR was performed in a total volume of 16 μL, which consisted of 2 μL of the cDNA template, 8 μL of 2× SYBR Green Master mix (TaKaRa, Japan), 0.3 μL of ROX reference dye II, 4.5 μL of PCR-grade water, and 0.6 μL (10 mM) of each primer. The running program was set as follows: 95°C for 30 s; followed by 40 cycles of 95°C for 5 s and annealing temperature 56°C for 32 s. At the end of reaction, PCR melting curve analysis was conducted to confirm single PCR products. The relative expression level of each candidate DEG was determined using the comparative 2^-ΔΔ^*^C^*^t^ method (Livak and Schmittgen, 2001). The concrete formula was as follows:

**Table 1:** Primers used in verification of RNA-Seq results by qRT-PCR.

Unigene ID	Gene name	Primer sequences (5′ → 3′)	Tm (°C)
Unigene17461_All	HEAT repeat-containing protein 5B-like	F: CAGAAGACCCTGTGGTGAAGTTG R: AAGAGGTAGCTGATGAGGATTGG	60.4
Unigene11885_All	Proto-oncogene tyrosine-protein kinase ROS-like	F:GATGGCAGCGTCTATCTTTCAGT R:CCAACCAGTATTTCCAGAACACG	60.9
Unigene34117_All	Rho GTPase-activating protein 17-like	F:AGTGACATTATGAGGCGGCAGAT R:CCTGAGCCGATAAGACATCCTGA	62.9
Unigene35151_All	ATP-dependent zinc metalloprotease YME1L1-like	F: TGGTCTTGCCCCTCCCTCACTTG R:TCCCTAAGATTACTTCCTTCGGT	63.9
Unigene13883_All	Heat shock 70 kDa protein IV-like	F:CAAATGACCCAAGCGAAGAAATC R:TTAGGGACGCCCCGAGGTGCAGG	68.8
Unigene38500_All	Ras association domain-containing protein 5-like isoform 2	F: TTCTAAAACGCATCGGATACGCT R:AGTCCAGTCCCACATCATTCAAG	61.9
Unigene38598_All	Reelin-like	F:GGAGCATCTACCACTCTGGAAGG R:TCTAACCCAAAGGCTGGAGCAAG	63.4
Unigene29644_All	Solute carrier organic anion transporter family member 4A1-like	F:CAGAGGAAAGAAAAGCAGCATAG R:GATGGGACTGTTACAGAACTGCA	59.0
Unigene51938_All	DNA replication licensing factor mcm5-like isoform 2	F:TAAGACCAAGGTGACTGTAGGAA R:TGATTTCATAGATGTTGGGATTG	56.7
Unigene3023_All	Calcium-activated chloride channel regulator 1-like	F:TGTTTACATCAGCATCTCAGCGTC R:CCCTTCTTTCCCACATCCTTCAA	63.2
Unigene32054_All	Transforming growth factor-beta-induced protein ig-h3-like	F:ATGAAGATGCTGATGTTGTTTGC R:AGGACCTCCAGTGTTGAGTGTTT	59.5
Unigene33033_All	Slit homolog 1 protein-like	F:AAGACAGGGACATCGTTGTTTTC R:AGCGTCCTTGGAAATGCTGGTTA	62.5

$$\begin{array}{c} \Delta \Delta Ct = [Ct(sample) - Ct(internal reference)] - [Ct(control)- Ct (internal reference)]. \end{array}$$

### Protein–Protein Interaction (PPI) Analysis

We used Blastx v2.2.28 (Zhang et al., 2000) with an *e*-value cutoff of 1e-10 to align the *S. intermedius* DEG sequences with the protein sequences from the sea urchin *S. purpuratus*. A PPI network was built using STRING^2^ (Szklarczyk et al., 2011) based on the PPI network of *S. purpuratus*. We used Cytoscape v3.5.1 (Shannon et al., 2003) to visualize the PPI network.

### Data Analysis

All of the data were expressed as the mean ± standard deviation (SD). All of the statistical analyses were performed with SPSS 16.0 (IBM, Shanghai, China). We first confirmed that our data were normally distributed and homogeneous with the Shapiro–Wilk test and with Levene’s test. We then compared differences in survival rate, SGR among treatments with one-way ANOVA (factor: temperature). We considered *p* < 0.05 as statistically significant and *p* < 0.01 as statistically extremely significant. Significant differences between pairs of treatments were identified with Duncan’s multiple range tests.

## Results

### The Impact of High Seawater Temperature on Growth, Survival, and Feeding of *S. intermedius*

**FIGURE 1:**
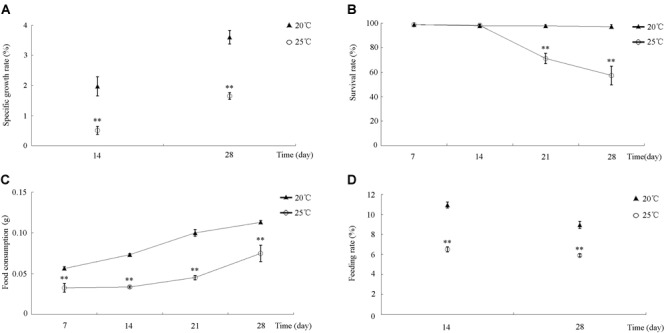
The effect of high temperature stress on SGR, SR, FC, and FR in *S. intermedius*. **(A)** The specific growth rates of *S. intermedius* cultured at 20 and 25°C. **(B)** The survival rates of *S. intermedius* between 20°C (control) and 25°C. **(C)** *S. intermedius* food consumption at 20 and 25°C. **(D)** *S. intermedius* feeding rates at 20 and 25°C. Values are expressed as the mean ± SD (*n* = 3). ^∗^Significant differences at *p* < 0.05 vs. control; ^∗∗^extremely significant differences at *p* < 0.01 vs. control.

During the 30-day incubation, differences in *S. intermedius* growth, survival, and feeding among all of the treatments were compared and analyzed statistically. *S. intermedius* reared at both 20 and 25°C exhibited an increase in SGR (Figure 1A), but the SGRs of *S. intermedius* reared at 25°C were significantly lower compared to those reared at 20°C. During the first 14 days of incubation, no significant difference in survival was observed between 20 and 25°C treatments, whereas survival rates decreased after 14 days of incubation at 25°C (Figure 1B). Among all of the experimental groups, food consumption increased in a time-dependent manner, whereas that in *S. intermedius* reared at 25°C was relatively lower compared to those reared at 20°C (Figure 1C). In addition, significantly reduced feeding rates were observed at 25°C compared to 20°C (Figure 1D).

### RNA Sequencing, Transcriptome Assembly, and Annotation

**FIGURE 2:**
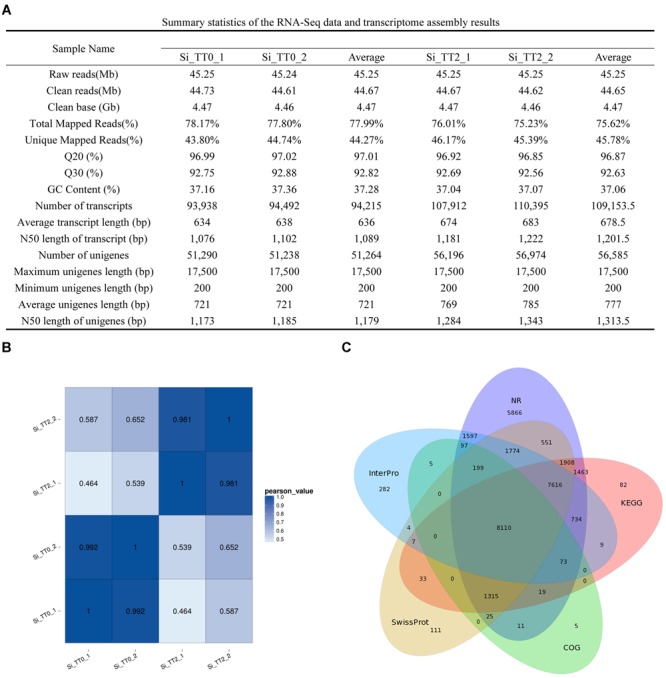
Summary statistics of the *S. intermedius* transcriptomes. **(A)** Summary statistics of the RNA-Seq data and transcriptome assembly results. **(B)** Pearson correlation coefficients between samples used in the current study. **(C)** Venn diagram showing NR, COG, KEGG, Swiss-Prot, and InterPro.

Four RNA sequencing (RNA-Seq) libraries (Si_TT0_1, Si_TT0_2, Si_TT2_1, and Si_TT2_2) from the intestines of *S. intermedius* cultured at 20 and 25°C were constructed and subsequently sequenced on an Illumina Hiseq 4000 platform. Approximately 45.24–45.25 million raw reads were obtained from each pooled sample (Figure 2A). After trimming, 44.61–44.73 million clean reads were obtained from each sample (Figure 2A). The Q20 range of all of the samples was 96.85–97.02%, and the Q30 range of all samples was 92.56–92.88%. The average GC content was 37.16 ± 0.14% (Figure 2A).

The transcriptome of *S. intermedius* was *de novo* assembled using the Trinity software with min_kmer_cov set to 2 by default, and all of the other parameters set to default. Completeness of the *de novo* assembly was assessed with BUSCO using a eukaryotic database^3^ of 303 genes. The assembled genes were deemed 82.5% complete by BUSCO (61.4% as single genes and 21.1% as duplicated genes). In the intestines of *S. intermedius* cultured at 20°C (Si_TT0), a total of 188,430 transcripts were obtained, with an average length of 636 bp and N50 length of 1,089 bp, and approximately 102,528 unigenes with a mean length of 721 bp and N50 length of 1,179 bp were also generated (Figure 2A). As for *S. intermedius* cultured at 25°C (Si_TT2), the intestine transcriptome analysis indicated that a total of 218,307 transcripts were obtained, with an average length of 678.5 bp and N50 length of 1201.5 bp, and approximately 113,170 unigenes with a mean length of 777 bp and N50 length of 1313.5 bp were generated as well (Figure 2A). In summary, approximately 46.33% of the transcripts in Si_TT0 and approximately 53.67% of the transcripts in Si_TT2 were successfully mapped back to the *de novo* transcriptome assembly, respectively (Figure 2A). Pearson’s correlation coefficients of FPKM distribution among the two biological replicates indicated the reproducibility of RNA-seq data (see Figure 2B). Annotation of assembled transcriptome was performed for comprehensive functional annotation of each unigene. After alignment, 65,349 unigenes were annotated in at least one of seven databases (Figure 2C).

### Identification of SNPs and SSRs

**FIGURE 3:**
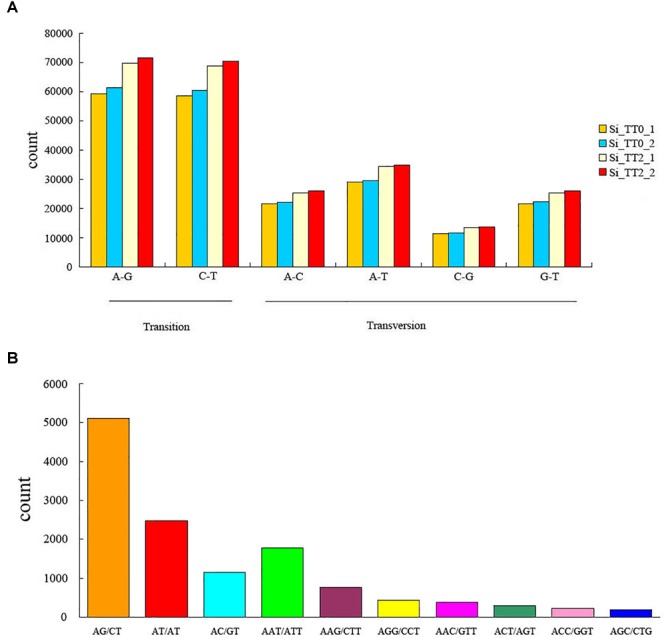
Frequency of identified **(A)** single nucleotide polymorphisms and **(B)** simple sequence repeats among the transcriptome libraries Si_TT0 (*n* = 2) and Si_TT2 (*n* = 2).

A total of 890,154 SNPs and 20,239 SSRs were identified from the assembled transcriptome. In SNPs, percent transition (Ts) and percent transversion (Tv) were 58.5 and 41.5%, respectively (Figure 3A). Ts was higher than Tv among the four libraries, and the Ts/Tv ratio was 117:83. The most common transitions were A–G (262,240, 29.46%) and C–T (258,526, 29.04%), and the predominant transversion type was A–T (128,089, 14.39%). The AG/CT repeat was the most abundant type of motif, the proportion gradient of repeat motifs in decreasing order was as follows: dimers (8,754, 43.25%) > monomers (5,573, 27.54%) > trimers (4,925, 24.33%) > pentamers (451, 2.22%) > quadmers (380, 1.88%) > hexamers (156, 0.77%) (Figure 3B).

### Analysis of Differentially Expressed Genes (DEGs)

**FIGURE 4:**
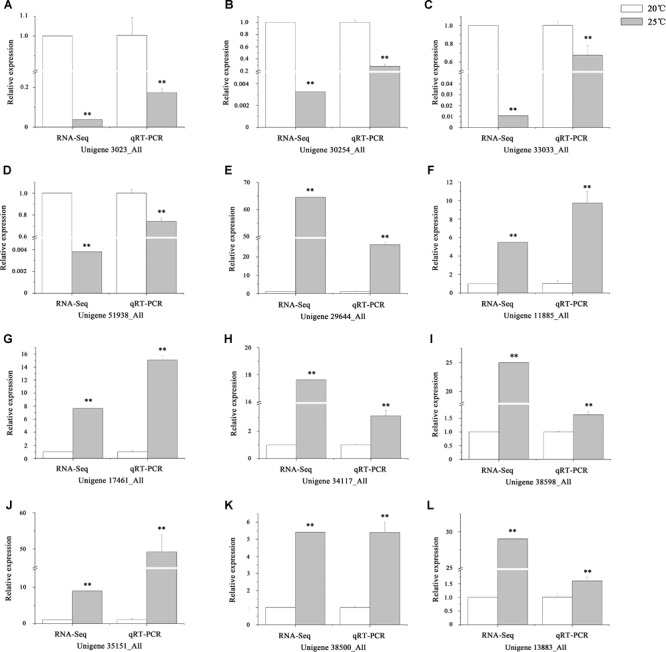
Verification of RNA-Seq by qRT-PCR. **(A–L)** The expression trends of 12 randomly selected DEGs. Values are expressed as the mean ± SD (*n* = 3). ^∗^Significant differences at *p* < 0.05 vs. control. ^∗∗^Extremely significant differences at *p* < 0.01 vs. control.

After removing the duplicated unigenes, approximately 59,846 genes expressed in the intestines of *S. intermedius* cultured at 20°C (Si_TT0) and 82,676 genes expressed in the intestines of *S. intermedius* cultured at 25°C (Si_TT2) were identified by Trinity with default parameters. DEGs between Si_TT0 (as control) and Si_TT2 were identified with a fold change ≥ 2.00 and probability ≥ 0.8. A total of 2,125 DEGs were identified, which included 1,015 upregulated and 1,110 downregulated unigenes as compared to those expressed in Si_TT0. Of the 2,125 DEGs, 732 genes were expressed specifically in Si_TT2, and 919 genes were expressed specifically in Si_TT0. qRT-PCR analysis indicated that the expression trends of 12 randomly selected DEGs (8 upregulated and 4 downregulated) were correlated well with those obtained in RNA-seq analysis, indicating the reliability and accuracy of the RNA-seq data obtained in this study (see Figure 4).

**FIGURE 5:**
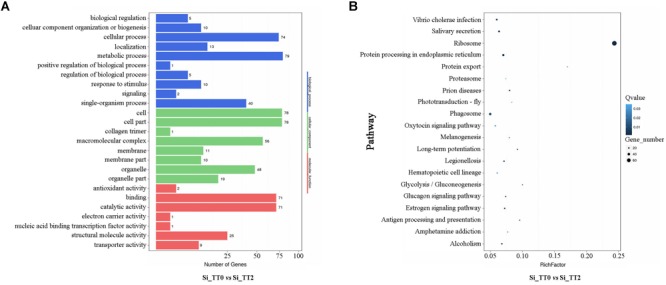
Functional annotation of DEGs of Si_TT2 vs. Si_TT0 in *S. intermedius.* **(A)** The most enriched GO terms of DEGs of *S. intermedius* Si_TT0and *S. intermedius* Si_TT2. The *Y*-axis represents the categories of annotated DEGs, and the *X*-axis represents the number of DEGs. **(B)** The top 20 enriched KEGG terms of DEGs of Si_TT2 vs. Si_TT0 in *S. intermedius*. The *Y*-axis represents the KEGG pathway, and the *X*-axis represents the enrichment factor. Dot size indicates the number of DEGs in the pathway. Dot colors corresponds to different *Q*-values.

All of the identified DEGs were then annotated with GO terms. Approximately 79, 78, and 78 DEGs were clustered as categories related to metabolic process, cell, and cell parts, respectively (see Figure 5A). A total of 2,125 identified DEGs were enriched in 247 pathways by KEGG analysis. The ribosome pathways were the most enriched pathways, and followed by protein processing in endoplasmic reticulum pathways, prion disease pathways, and protein export pathways (see Figure 5B). Several DEGs related to growth (Supplementary Table S1), energy metabolism (Supplementary Table S2), heat shock responses (Supplementary Table S3), and immune response (Supplementary Table S4) were identified.

**FIGURE 6:**
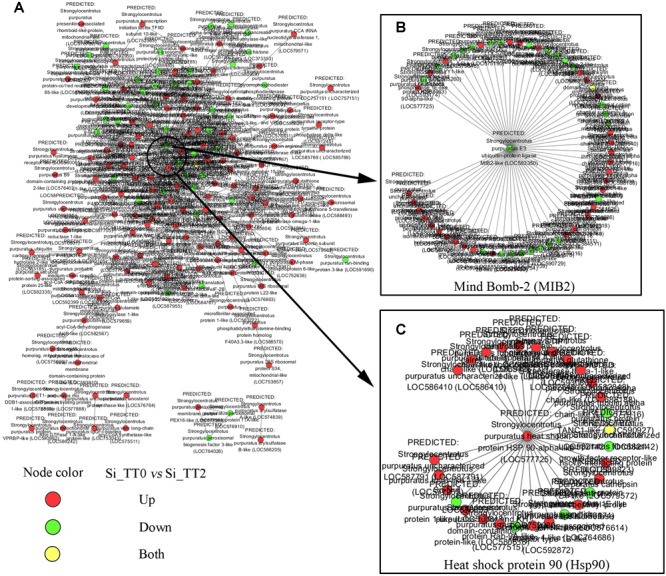
Differentially expressed genes interactive network prediction. **(A)** Interactive network prediction of all DEGs. **(B)** Interactive network prediction of MIB2. **(C)** Interactive network prediction of heat shock protein 90.

For further elucidate interaction relationships, prediction analysis was conducted. After examining PPI networks, 173 proteins were found to be extremely well connected. In the 25°C treatments, 127 proteins were upregulated, 45 proteins were downregulated compared to the 20°C treatments (Figure 6A). These well-connected proteins included E3 ubiquitin-protein ligase MIB2 (GenBank Accession Number gi| 390364157), with 58 connections (Figure 6B); and heat shock protein 90 (GenBank Acc. No. gi| 390340697), with 29 connections (Figure 6C).

**FIGURE 7:**
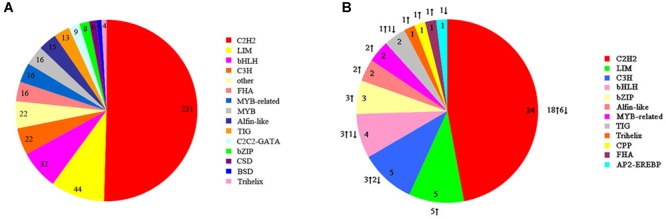
Transcription factor families identified in *S. intermedius* Si_TT0 and *S. intermedius* Si_TT2 transcriptomes. **(A)** The distribution of all of the identified transcription factors. The number of each transcription factor is shown. **(B)** The distribution of differentially expressed transcription factors. The number and the expression of each differentially expressed transcription factor are shown.

Transcription factors are also key regulators involved in heat-stress response in organisms. We identified 458 potential TFs from 25 TF families in *S. intermedius* cultured at 20 and 25°C. The identified TFs most commonly belonged to the following families: Cys2His2 protein (C2H2; 231 TFs; 50.44%), LIM domain-containing protein (LIM; 44 TFs; 9.61%), basic-helix-loop-helix protein (bHLH; 32 TFs; 6.99%), CCCH-type zinc finger protein (C3H; 22 TFs; 4.80%), and the basic leucine zipper protein (bZIP; 8 TFs; 1.75%) (Figure 7A). In the 25°C treatments, 40 TFs were upregulated and 11 TFs were downregulated compared to the 20°C treatments (Figure 7B and Supplementary Table S5).

## Discussion

In the present study, we first investigated the impact of high water temperature on the growth, survival, and feeding behavior of *S. intermedius*. As we expected, high seawater temperature imparted significantly negative effects on the survival, growth, and feeding of *S. intermedius*. These results support the opinion that the growth, survival and feeding of sea urchins are sensitive to temperature (Siikavuopio et al., 2008; Pearce et al., 2010; Onitsuka et al., 2013). Moreover, we found that the lower SGRs of *S. intermedius* suffered from high seawater temperature stress were due to a decrease in feeding rates in this study, which agrees with the findings of Yu et al. (2016), who reported that high seawater temperature reduces the feeding and SGRs of sea cucumber *A. japonicus*. The observed decrease in food consumption and feeding rate not only explains the slow growth of *S. intermedius*, but also reflects to some extent the decrease in energy budget of *S. intermedius* under high temperature stress. This observation is also consistent with the results of Watts et al. (2011).

Organisms undergo metabolic adjustments in the presence of environmental stimuli. Moreover, cellar stress responses (CSRs) can also reflect the responses of an individual to environmental fluctuations (such as chronic high seawater temperature stress in this study) at the cellular and tissue levels. We therefore subsequently investigated the molecular mechanisms underlying high temperature-driven decrease in growth, survival, and feeding in *S. intermedius* by constructing four high-quality RNA-seq libraries and performing comparative transcriptome analysis to identify gene candidates associated with high-temperature responses.

In terms of the decreased energy budget, our data indicated that the expression of ATP5 [δ or the oligomycin sensitivity-conferring protein (OSCP)] mRNA significantly decreased in *S. intermedius* cultured at 25°C compared to those incubated at 20°C. ATP5 is one of subunits of ATP synthase, and silencing the expression of ATP5 can affect the activity of ATP synthase and further block the electron transfer chain in organisms. Our data suggest that high temperature stress can block ATP synthesis and affect the energy charge by decreasing *atp*5 expression in sea urchins. A decrease in ATP5 expression can alter total cellular ATP levels and impair growth in plants (Robison et al., 2009), which bears some resemblance to our observations. However, we could not find any studies involving echinoderm ATP5 to compare our results with. In addition to a reduction in energy, another other common strategy for organisms to adapt environmental stress (e.g., warming in this study) involves decreasing energetically expensive metabolic processes to extend their duration of tolerance (Pörtner and Farrell, 2008). Histones are dynamic proteins that can undergo multiple types of post-translational modifications and regulate gene expression depending on the metabolic state of the cell. Padilla-Gamino et al., 2013 reported that the downregulation histone-encoding genes is a principal transcriptional response accompanying metabolic depression in *S. purpuratus* larvae cultured at higher temperature (18°C), and that histones possibly act as metabolic sensors. We also observed a reduction in the expression of histone-encoding genes, such as Unigene17018_All (fold change: -2.42), Unigene9687_All (fold change: -7.70), and Unigene13915_All (fold change: -8.64). These findings support the hypothesis that sea urchins exposed to higher temperature consume energy supplies more rapidly than those cultured at optimal temperatures by reducing key metabolites for histone-modifying enzymes. In addition, another strategy for organisms to adapt environmental stress is minimizing body size or delaying growth. In this study, we found a significant reduction in expression of transforming growth factor beta (TGF-β) transcripts. TGF-β acts as a cytokine by imparting immunoregulatory effects, including lymphocyte proliferation, cytokine responsiveness, or cytokine expression (Ruscetti and Palladino, 1991). In echinoderms, TGF-β plays an important role in the symmetrical growth of sea urchin embryos and the biomineralization of larval skeletogenesis (Zito et al., 2003). In this study, we postulate that the reduced growth under high temperature stress in *S. intermedius* might be due to alterations in TGF signals. Further studies should be conducted to clarify the mechanisms underlying how high-temperature influences the TGF signal pathway.

Heat-shock proteins (HSPs) are molecular chaperones with multiple functions, including stress resistance and adaption to environmental changes in various species (Currie, 2011). The upregulation of Hsps is one of ubiquitous mechanisms of marine organism in coping with thermal stress (Osovitz and Hofmann, 2005). Our data also identified altered mRNA expression levels of some Hsps. Runcie et al. (2012) demonstrated that higher temperatures (18°C vs. 12°C) can cause mild embryonic developmental stress and increase both *hsp*70 and *hsp*90 expression in embryos of the sea urchin *S. purpuratus*. Vergara-Amado et al. (2017) reported that transient warmer temperature treatments (18°C vs. 14°C) induces the up-regulation of *hsp*70 and *hsp*90 in juveniles of the sea urchin *L. albus*. As expected, we found the upregulation of *hsp*70 and *hsp*90 in adult *S. intermedius* after application of chronic high-temperature stress. Hsp70 and Hsp90 have both been implicated in the proteasomal degradation of chaperoned client proteins (Kiang and Tsokos, 1998; Pratt, 1998). Hsp90, in particular, has been characterized as the driver for Hsp-mediated proteasomal degradation. Hsp90 is a protective chaperone when in complex with p50/immunophilin, p23, and ATP, but drives client proteins to poly-ubiquitination and proteasomal degradation when ATP-depleted and bound in complex with Hsp70 (Doong et al., 2003). Interestingly, the present study observed that *mind bomb-2* (*mib2*) was downregulated under high temperature (25°C) conditions as compared to that observed using the optimal temperature (20°C). MIB2 has E3 ubiquitin-protein ligase activity and can promote the ubiquitination and endocytosis of its client protein (Koo et al., 2005). PPI prediction indicated the upregulation of Hsp90 and the downregulation of E3 ubiquitin-protein ligase MIB2 under high temperature (25°C) conditions as compared to that observed using the optimal temperature (20°C) (Figure 6B). This observation suggests that high temperature-induced proteasomal degradation or apoptosis might mainly rely on the Hsp-mediated proteasomal degradation pathway rather than that of being MIB-2-mediated. In addition, the cytokine-like function of Hsp70 what been well documented in several studies (Zhang et al., 2011; Yang et al., 2016; Ying et al., 2016). Combined with the findings of the present study, these observations support the hypothesis that Hsp70 and Hsp90 regulate not only proteasomal degradation but also immune responses when sea urchins are subjected to high-temperature stress (Ying et al., 2016). Since enhanced *hsp*70 and *hsp*90 transcripts can be detected from embryos to adults in sea urchins during high temperature stress regardless of whether such exposure is chronic or transient, we therefore hypothesize that the *hsp*70 and *hsp*90 genes be included in selective breeding or assisted breeding of high temperature-resistant sea urchins.

The significant upregulation of the glutathione *S*-transferases (GST) gene was also observed in the present study. GST is a phase II detoxification isozyme that catalyzes the conjugation of glutathione with both xenobiotics and endogenous substrates. GST activity has long been utilized as a bioindicator of environmental contamination in coastal regions (Cunha et al., 2005). Field studies have shown that the maximal GST activity in sea urchins and mussels can be observed in the summer (Moreira and Guilhermino, 2005). However, no study has measured echinoderm GST levels under thermal-stress, and thus we were unable to clarify the relationship between GST activities and high temperature stress in sea urchins.

Transcription factors and *cis*-acting elements are conserved mechanisms that regulate gene transcription (Murray et al., 1988). Among differentially expressed TFs, we observed the significantly upregulation of mRNA expression of some multiple function TFs in sea urchins in response to high temperature stress, which include activator protein-1 (AP-1), FOS, cAMP-response element-binding protein (CREB), and the zinc finger (ZNF) proteins. AP-1 and FOS are members of the basic leucine zipper protein (bZIP) family. Activated AP-1 has been demonstrated to be a stress-responsive TF and plays a key role in responding to environmental stimuli by regulating various immune signal transduction pathways, such as the Toll-like receptor (TLR), tumor necrosis factor alpha (TNF-α), and mitogen-activated protein kinase (MAPK) pathways, in marine organisms (Qu et al., 2015; Zhan et al., 2018). Fos proteins are a key part of the AP-1 complex and can regulate a wide range of biological process (Hirayama et al., 2005). Production of many immune-related molecules (antioxidant enzymes, chemokines, and interleukin) require Fos expression (Rimoldi et al., 2009). Additionally, it has been shown that Fos cooperates with Notch to regulate cell fate specification of intermediate precursors during *Caenorhabditis elegans* development (Oommen and Newman, 2007). CREB is one of multi-function TF regulating various signal transduction pathways. In *Drosophila*, CREB and Hsp70 can additively suppress polyglutamine-mediated toxicity (Iijimaando et al., 2005). In cultured rat primary hippocampal neuron cells, CREB has been demonstrated to be activated by the Hsp90/Akt signal pathway (Cen et al., 2006). Combined with our comparative expression data on *hsp70 and hsp90*, we postulate that the CREB-mediated gene regulation network of higher thermal tolerance in *S. intermedius* might be closely correlated with HSPs. Further studies should be conducted to confirm this hypothesis. ZNFs can be found in all eukaryotes and act as TFs that plays critical role in responding to environmental stimuli such as biometals (Villalpando et al., 2017). In plants, ZNFs have been identified as heat response-related gene candidates (Wang et al., 2010; Yan et al., 2016; Fang et al., 2017). The present study has shown that ZNFs are involved in echinoderm heat responses and more studies should be conducted to elucidate the ZNF regulated genes and signal transduction pathways during chronic heat stress in echinoderms.

Moreover, the adjustment of gene structure is the ultimate mechanism for organisms to adapt to long-term stress and maintain population size. Our comparative transcriptomic data also indicated altered SNPs and SSRs. These results will facilitate studies on the genetic structure, population geography, and ecology of sea urchins. Further screening of these SNPs and SSRs may assist in the identification of more valuable heat-resistant genetic markers that may be utilized in the selective breeding of heat-resistant sea urchins.

However, significant expression alterations of some transient thermal-tolerance candidate genes such as cytochrome P450 (CYP450), Na^+^/K^+^ ATPase (Vergara-Amado et al., 2017) were not observed in the present study. This suggests that high temperature-induced molecular responses in the same species depend on individual developmental stages and the duration of stress.

## Conclusion

The present study has demonstrated that chronically high seawater temperatures negatively influence the growth, survival, and feeding of the sea urchin *S. intermedius*. Gene candidates (e.g., HSPs, cytokines, and TFs) that were closely correlated with thermal resistance and adaptation were identified by comparative transcriptomic analysis. In summary, our results provide insight into the genes and regulatory networks involved in chronic thermal stress in *S. intermedius*, as well as enriching transcriptomic and genetic resources for sea urchins and other invertebrates. More studies on the molecular events involved in the thermal resistance and adaption mechanisms for *S. intermedius* should be conducted to better understand the impact of chronically high temperature stress on sea urchin physiology and ecology.

## Data Availability

The datasets generated for this study can be found in NCBI, PRJNA508827.

## Author Contributions

YC and YZ conceived and designed the experiments. JL, JS, WZ, YL, DC, and WH performed the experiments. JL, YZ, and WZ analyzed the data. YZ and JL wrote the manuscript. All authors read and approved the manuscript.

## Conflict of Interest Statement

The authors declare that the research was conducted in the absence of any commercial or financial relationships that could be construed as a potential conflict of interest.

## Footnotes

1 http://pgrc.ipk-gatersleben.de/misa/misa.html

2 http://string-db.org/

3 http://busco.ezlab.org/datasets/eukaryota_odb9.tar.gz
