# Supplementary material for: The Impact of Chronic Heat Stress on the Growth, Survival, Feeding, and Differential Gene Expression in the Sea Urchin Strongylocentrotus intermedius
Source: Front Genet. 2019 Apr 4;10:301. doi: 10.3389/fgene.2019.00301 (PMC6458246; doi:10.3389/fgene.2019.00301)
Supplement: Supplementary file 1 [file Table_1.DOC]

**Table S1 Summary of growth-related genes that were specifically expressed in Si_TT2 *vs.* Si_TT0**

| Unigene | Unigene expression | | log_2_(fold-change)  Si_TT2/Si_TT0 | Description |
| --- | --- | --- | --- | --- |
|  | Si_TT0 | Si_TT2 |  |  |
| Unigene1769_All | 0.01 | 2.08 | 7.70 | Coagulation factor II |
| Unigene49686_All | 0.01 | 1.86 | 7.54 | Collagen alpha-2(V) chain-like |
| CL5492.Contig2_All | 0.01 | 1.77 | 7.46 | Neurogenic locus Notch protein |
| Unigene39213_All | 0.01 | 1.64 | 7.36 | Protein VPRBP |
| Unigene10763_All | 33.74 | 225.53 | 2.74 | Neurocan core protein |
| CL8293.Contig2_All | 5.62 | 32.57 | 2.53 | GTPase KRas |
| Unigene12047_All | 19.22 | 109.51 | 2.51 | Receptor-type tyrosine-protein phosphatase F |
| Unigene7658_All | 149.76 | 22.91 | -2.71 | Deleted in malignant brain tumors 1 protein-like |
| CL8796.Contig1_All | 108.41 | 14.83 | -2.87 | Fibulin 1/2 |
| CL8796.Contig2_All | 96.69 | 9.96 | -3.28 | Fibrillin-2-like |
| Unigene30722_All | 9.35 | 0.92 | -3.35 | Adhesion G protein-coupled receptor E5 |
| Unigene3111_All | 44.9 | 3.51 | -3.68 | Mucin 2, oligomeric mucus/gel-forming |
| Unigene7176_All | 17.14 | 0.32 | -5.77 | Neurogenic locus Notch protein-like |
| Unigene31179_All | 1.67 | 0.01 | -7.38 | Isocitrate dehydrogenase (NAD+) |

Table S1. Continued on next page

Table S1. Continued.

| Unigene | Unigene expression | | log_2_(fold-change)  Si_TT2/Si_TT0 | Description |
| --- | --- | --- | --- | --- |
|  | Si_TT0 | Si_TT2 |  |  |
| Unigene22107_All | 1.87 | 0.01 | -7.55 | Collagen alpha-1(IV) chain-like |
| Unigene13673_All | 2.06 | 0.01 | -7.68 | Tartrate-resistant acid phosphatase type 5 |
| Unigene31433_All | 2.54 | 0.01 | -7.98 | NOTCH2 |
| Unigene21170_All | 2.9 | 0.01 | -8.18 | Latent transforming growth factor beta binding protein 4 |
| Unigene23497_All | 3.44 | 0.01 | -8.43 | Mitogen-activated protein kinase 1-like |
| Unigene13151_All | 4.94 | 0.01 | -8.95 | FBN1 |
| Unigene20011_All | 5.12 | 0.01 | -9 | Collagen alpha-1(XII) chain-like |
| Unigene51189_All | 5.57 | 0.01 | -9.12 | Notch |
| Unigene9662_All | 10.82 | 0.01 | -10.08 | NOTCH4 |
| Unigene24201_All | 11.46 | 0.01 | -10.16 | Cofilin-2-like |
