# Supplementary material for: The Impact of Chronic Heat Stress on the Growth, Survival, Feeding, and Differential Gene Expression in the Sea Urchin Strongylocentrotus intermedius
Source: Front Genet. 2019 Apr 4;10:301. doi: 10.3389/fgene.2019.00301 (PMC6458246; doi:10.3389/fgene.2019.00301)
Supplement: Supplementary file 2 [file Table_2.DOC]

**Table S2 Summary of energy metabolism-related genes that were specifically expressed in Si_TT2 *vs.* Si_TT0*.***

| Unigene | Unigene expression | | log_2_(fold-change)  Si_TT2/Si_TT0 | Description | |
| --- | --- | --- | --- | --- | --- |
|  | Si_TT0 | Si_TT2 |  |  |  |
| CL3317.Contig2_All | 196.11 | 25.82 | -2.92 | Sulfur dioxygenase |  |
| Unigene21333_All | 1.59 | 0.01 | -7.31 | Ubiquinol-cytochrome c reductase subunit 7 |  |
| Unigene16115_All | 1.61 | 0.01 | -7.33 | V-type H^+^-transporting ATPase subunit A |  |
| Unigene11760_All | 1.70 | 0.01 | -7.41 | V-type H^+^-transporting ATPase subunit G |  |
| Unigene21687_All | 1.86 | 0.01 | -7.54 | V-type H^+^-transporting ATPase subunit B |  |
| Unigene26212_All | 1.88 | 0.01 | -7.56 | Cytochrome c oxidase subunit 6b |  |
| Unigene20900_All | 1.90 | 0.01 | -7.57 | F-type H^+^-transporting ATPase subunit alpha |  |
| Unigene18044_All | 2.13 | 0.01 | -7.73 | V-type H^+^-transporting ATPase subunit E |  |
| Unigene11244_All | 2.21 | 0.01 | -7.79 | Inorganic pyrophosphatase |  |
| Unigene1818_All | 2.82 | 0.01 | -8.14 | F-type H^+^-transporting ATPase subunit c |  |
| Unigene21172_All | 2.94 | 0.01 | -8.20 | 3'-Phosphoadenosine 5'-phosphosulfate synthase |  |
| Unigene15961_All | 3.24 | 0.01 | -8.34 | 5 V-Type H^+^-transporting ATPase 16kDa proteolipid subunit |  |
