# Supplementary material for: The Impact of Chronic Heat Stress on the Growth, Survival, Feeding, and Differential Gene Expression in the Sea Urchin Strongylocentrotus intermedius
Source: Front Genet. 2019 Apr 4;10:301. doi: 10.3389/fgene.2019.00301 (PMC6458246; doi:10.3389/fgene.2019.00301)
Supplement: Supplementary file 3 [file Table_3.DOC]

**Table S3 Summary of heat shock protein family-related genes that were specifically expressed in Si_TT2 *vs* Si_TT0*.***

| Unigene | Unigene expression | | log_2_(fold-change)  Si_TT2/Si_TT0 | Description | |
| --- | --- | --- | --- | --- | --- |
|  | Si_TT0 | Si_TT2 |  |  |  |
| CL6332.Contig2_All | 0.01 | 8.33 | 9.70 | Heat shock protein HSP 90-alpha 1-like |  |
| CL6332.Contig3_All | 0.17 | 7.99 | 5.60 | Heat shock protein HSP 90-alpha 1-like |  |
| CL8887.Contig1_All | 1.69 | 76.47 | 5.50 | Heat shock 70 kDa protein IV-like |  |
| Unigene13883_All | 0.25 | 7.12 | 4.86 | Heat shock 70 kDa protein IV-like |  |
| CL8887.Contig2_All | 3.97 | 80.33 | 4.34 | heat shock 70 kDa protein IV-like |  |
| Unigene24893_All | 22.23 | 231.64 | 3.38 | Heat shock protein HSP 90-alpha 1-like |  |
| Unigene596_All | 15.42 | 131.12 | 3.09 | 10-kDa Heat shock protein, mitochondrial-like |  |
| Unigene14867_All | 30.97 | 192.65 | 2.64 | 60-kDa Heat shock protein, mitochondrial-like |  |
