# Supplementary material for: The Impact of Chronic Heat Stress on the Growth, Survival, Feeding, and Differential Gene Expression in the Sea Urchin Strongylocentrotus intermedius
Source: Front Genet. 2019 Apr 4;10:301. doi: 10.3389/fgene.2019.00301 (PMC6458246; doi:10.3389/fgene.2019.00301)
Supplement: Supplementary file 4 [file Table_4.DOC]

**Table S4 Summary of immune response-related genes that were specifically expressed in Si_TT2 *vs.* Si_TT0*.***

| Unigene | Unigene expression | | log_2_(fold-change)  Si_TT2/Si_TT0 | Description | |
| --- | --- | --- | --- | --- | --- |
|  | Si_TT0 | Si_TT2 |  |  |  |
| CL4894.Contig1_All | 0.01 | 3.22 | 8.33 | Immunoglobulin superfamily member 10-like |  |
| CL4378.Contig4_All | 0.17 | 26.17 | 7.31 | CD151 antigen |  |
| Unigene50776_All | 0.01 | 1.54 | 7.27 | CUB and sushi domain-containing protein |  |
| CL8887.Contig1_All | 1.69 | 76.47 | 5.50 | L1 cell adhesion molecule like protein |  |
| Unigene50382_All | 0.24 | 6.48 | 4.78 | Complement factor H |  |
| CL7960.Contig1_All | 0.97 | 10.84 | 3.49 | Interferon induced transmembrane protein |  |
| Unigene24893_All | 22.23 | 231.64 | 3.38 | Molecular chaperone HtpG |  |
| Unigene5929_All | 1.75 | 15.40 | 3.14 | Large subunit ribosomal protein L35 |  |
| CL1070.Contig1_All | 6.27 | 43.93 | 2.81 | Calreticulin |  |
| CL8293.Contig2_All | 5.62 | 32.57 | 2.53 | GTPase KRas |  |
| Unigene8399_All | 61.88 | 11.08 | -2.48 | Integrin beta 3 |  |
| Unigene6627_All | 58.67 | 9.85 | -2.58 | Fc receptor, IgE, low affinity II |  |
| CL8796.Contig1_All | 108.41 | 14.83 | -2.87 | Fibulin 1/2 |  |
| CL8796.Contig2_All | 96.69 | 9.96 | -3.28 | Fibulin 1/2 |  |

Table S4. continued on next page

Table S4. Continued.

| Unigene | Unigene expression | | log_2_(fold-change)  Si_TT2/Si_TT0 | Description | |  |
| --- | --- | --- | --- | --- | --- | --- |
|  | Si_TT0 | Si_TT2 |  |  |  |  |
| CL6552.Contig1_All | 145.42 | 12.36 | -3.56 | ADP-ribosyl cyclase 1 |  |  |
| CL6552.Contig3_All | 116.94 | 9.44 | -3.63 | ADP-ribosyl cyclase 1 |  |  |
| Unigene10011_All | 48.19 | 3.28 | -3.88 | Wiskott-Aldrich syndrome protein |  |  |
| Unigene31179_All | 1.67 | 0.01 | -7.38 | Isocitrate dehydrogenase (NAD+) |  |  |
| Unigene25381_All | 2.01 | 0.01 | -7.65 | Myosin I |  |  |
| Unigene31995_All | 2.11 | 0.01 | -7.72 | Serine/threonine-protein phosphatase PP1 catalytic subunit |  |  |
| Unigene11222_All | 2.24 | 0.01 | -7.80 | Amphiphysin |  |  |
| Unigene15232_All | 2.32 | 0.01 | -7.85 | Aminopeptidase N |  |  |
| Unigene21170_All | 2.90 | 0.01 | -8.18 | Latent transforming growth factor beta binding protein 2/3/4 |  |  |
| Unigene32054_All | 3.07 | 0.01 | -8.26 | Transforming growth factor-beta-induced protein |  |  |
| Unigene23497_All | 3.44 | 0.01 | -8.43 | Mitogen-activated protein kinase 1/3 |  |  |
| CL5222.Contig1_All | 3.53 | 0.01 | -8.46 | Platelet/endothelial cell adhesion molecule |  |  |
| Unigene13151_All | 4.94 | 0.01 | -8.95 | Fibrillin 1 |  |  |
| Unigene20011_All | 5.12 | 0.01 | -9.00 | Collagen, type XII, alpha |  |  |

Table S4. Continued on next page

Table S4. Continued.

| Unigene | Unigene expression | | log_2_(fold-change)  Si_TT2/Si_TT0 | Description | |  |
| --- | --- | --- | --- | --- | --- | --- |
|  | Si_TT0 | Si_TT2 |  |  |  |  |
| CL4872.Contig1_All | 5.57 | 0.01 | -9.12 | Cathepsin L |  |  |
| Unigene1722_All | 6.73 | 0.01 | -9.39 | Molecular chaperone HtpG |  |  |
| CL5108.Contig1_All | 8.78 | 0.01 | -9.78 | L1 cell adhesion molecule like protein |  |  |
| Unigene26224_All | 9.44 | 0.01 | -9.88 | RNA-binding protein FUS |  |  |
| Unigene24201_All | 11.46 | 0.01 | -10.16 | Cofilin |  |  |
| Unigene11817_All | 17.58 | 0.01 | -10.78 | WAS protein family, member 2 |  |  |
