# Supplementary material for: The Impact of Chronic Heat Stress on the Growth, Survival, Feeding, and Differential Gene Expression in the Sea Urchin Strongylocentrotus intermedius
Source: Front Genet. 2019 Apr 4;10:301. doi: 10.3389/fgene.2019.00301 (PMC6458246; doi:10.3389/fgene.2019.00301)
Supplement: Supplementary file 5 [file Table_5.DOC]

**Table S5 Transcriptional factors up- or downregulated- in Si_TT2 relative to Si_TT0. FPKM: expected number of fragments per kilobase of transcript sequence per million base pairs sequenced.**

| Family | Unigene | NR description | KEGG description | Si_TT2 *vs.* Si_TT0 | Si_TT0 FPKM | Si_TT2 FPKM |
| --- | --- | --- | --- | --- | --- | --- |
| Alfin-like | Unigene46254_All | Pygopus homolog 2-like [*Amphimedon queenslandica*] | SHC-transforming protein 1 | UP | 0 | 2.10 |
|  | CL946.Contig2_All | Uncharacterized protein LOC100890719 [*Strongylocentrotus purpuratus*] | tRNA Nucleotidyltransferase (CCA-adding enzyme) | UP | 0.32 | 1.35 |
| AP2-EREBP | Unigene31500_All | Hypothetical protein, conserved, partial [*Eimeria maxima*] | - | DOWN | 1.01 | 0 |
| bHLH | Unigene27610_All | uncharacterized protein LOC575443 [*Strongylocentrotus purpuratus*] | Musculin (activated B-cell factor-1) | UP | 0 | 0.62 |
|  | Unigene19759_All | Transcription factor achaete/acute [*Hemicentrotus pulcherrimus*] | Achaete-scute complex protein | UP | 0.29 | 1.38 |
|  | Unigene10652_All | Transcription factor HES-4-like [*Strongylocentrotus purpuratus*] | Hairy and enhancer of split 1 | UP | 1.80 | 7.34 |
|  | CL900.Contig1_All | Uncharacterized protein LOC581970 [*Strongylocentrotus purpuratus*] | Hairy and enhancer of split 1 | DOWN | 4.46 | 1.02 |
| bZIP | Unigene12776_All | Transcription factor AP-1-like [*Strongylocentrotus purpuratus*] | Transcription factor AP-1 | UP | 38.86 | 193.59 |
|  | CL3674.Contig5_All | Cyclic AMP-responsive element-binding protein 3-like protein 3-like [*Strongylocentrotus purpuratus*] | Cyclic AMP-responsive element-binding protein 3 | UP | 13.87 | 66.93 |

Table S5. Continued on next page.

| Family | Unigene | NR description | KEGG description | Si_TT2 *vs.* Si_TT0 | Si_TT0 FPKM | Si_TT2 FPKM |
| --- | --- | --- | --- | --- | --- | --- |
|  | Unigene12517_All | Transcription factor kayak-like [*Strongylocentrotus purpuratus*] | Fos-like antigen, invertebrate | UP | 1.780 | 8.64 |
| C2H2 | Unigene9352_All | Zinc finger protein 227-like [*Strongylocentrotus purpuratus*] | KRAB Domain-containing zinc finger protein | UP | 0 | 1.56 |
|  | Unigene49656_All | Zinc finger protein 808-like isoform 1 [*Strongylocentrotus purpuratus*] | KRAB Domain-containing zinc finger protein | UP | 0 | 1.47 |
|  | Unigene11892_All | Uncharacterized protein LOC100889161 [*Strongylocentrotus purpuratus*] | - | UP | 0 | 0.89 |
|  | Unigene21842_All | Uncharacterized protein LOC100888504 isoform 2 [*Strongylocentrotus purpuratus*] | KRAB Domain-containing zinc finger protein | UP | 0 | 0.79 |
|  | Unigene40319_All | Uncharacterized protein LOC592869 [*Strongylocentrotus purpuratus*] | KRAB Domain-containing zinc finger protein | UP | 0 | 0.63 |
|  | CL4621.Contig2_All | Zinc finger protein 84-like [*Strongylocentrotus purpuratus*] | KRAB Domain-containing zinc finger protein | UP | 0 | 0.62 |

Table S5. Continued.

Table S5. Continued on next page.

| Family | Unigene | NR description | KEGG description | Si_TT2 *vs.* Si_TT0 | Si_TT0 FPKM | Si_TT2 FPKM |
| --- | --- | --- | --- | --- | --- | --- |
|  | Unigene39817_All | Zinc finger protein 271-like [*Strongylocentrotus purpuratus*] | KRAB Domain-containing zinc finger protein | UP | 0 | 0.60 |
|  | Unigene44100_All | Zinc finger protein 850-like [*Strongylocentrotus purpuratus*] | - | UP | 0 | 0.56 |
|  | Unigene41563_All | Uncharacterized protein LOC100891822 [*Strongylocentrotus purpuratus*] | KRAB Domain-containing zinc finger protein | UP | 0 | 0.49 |
|  | Unigene22318_All | Uncharacterized protein LOC100888350 [*Strongylocentrotus purpuratus*] | KRAB Domain-containing zinc finger protein | UP | 0.12 | 2.24 |
|  | Unigene39471_All | Histone-lysine N-methyltransferase PRDM9-like [*Strongylocentrotus purpuratus*] | PR Domain zinc finger protein 4 | UP | 0.06 | 0.42 |
|  | Unigene16049_All | Endothelial zinc finger protein induced by tumor necrosis factor alpha-like, partial [*Strongylocentrotus purpuratus*] | KRAB Domain-containing zinc finger protein | UP | 0.45 | 2.97 |
|  | Unigene28560_All | Uncharacterized protein LOC579988 [*Strongylocentrotus purpuratus*] | KRAB Domain-containing zinc finger protein | UP | 0.27 | 1.76 |

Table S5. Continued.

Table S5. Continued on next page.

| Family | Unigene | NR description | KEGG description | Si_TT2 *vs.* Si_TT0 | Si_TT0 FPKM | Si_TT2 FPKM |
| --- | --- | --- | --- | --- | --- | --- |
|  | Unigene12517_All | Transcription factor kayak-like [*Strongylocentrotus purpuratus*] | Fos-like antigen, invertebrate | UP | 1.80 | 8.64 |
|  | Unigene134_All | Zinc finger protein 85-like [*Strongylocentrotus purpuratus*] | KRAB Domain-containing zinc finger protein | UP | 0.19 | 1.13 |
|  | Unigene9608_All | Uncharacterized protein LOC580897 [*Strongylocentrotus purpuratus*] | IKAROS family zinc finger protein | UP | 0.22 | 1.25 |
|  | Unigene11689_All | Zinc finger protein 84-like [*Strongylocentrotus purpuratus*] | KRAB Domain-containing zinc finger protein | UP | 0.47 | 2.24 |
|  | CL3219.Contig7_All | Gastrula zinc finger protein xFG20-1-like [*Strongylocentrotus purpuratus*] | KRAB Domain-containing zinc finger protein | UP | 0.38 | 1.68 |
|  | Unigene14022_All | Uncharacterized protein LOC100889528 [*Strongylocentrotus purpuratus*] | Krueppel-like factor 6/7 | UP | 0.39 | 1.58 |
|  | Unigene16694_All | Zinc finger protein 84-like [*Strongylocentrotus purpuratus*] | KRAB Domain-containing zinc finger protein | DOWN | 1.66 | 0.40 |
|  | Unigene44484_All | Uncharacterized protein LOC580177 [*Strongylocentrotus purpuratus*] | - | DOWN | 0.74 | 0.14 |

Table S5. Continued.

Table S5. Continued on next page

| Family | Unigene | NR description | KEGG description | Si_TT2 *vs.* Si_TT0 | Si_TT0 FPKM | Si_TT2 FPKM |
| --- | --- | --- | --- | --- | --- | --- |
|  | Unigene31769_All | Zinc finger protein 91-like [*Strongylocentrotus purpuratus*] | KRAB Domain-containing zinc finger protein | DOWN | 2.22 | 0.37 |
|  | Unigene136_All | Zinc finger protein 85-like [*Strongylocentrotus purpuratus*] | KRAB Domain-containing zinc finger protein | DOWN | 1.16 | 0.17 |
|  | CL4509.Contig3_All | Histone-lysine N-methyltransferase PRDM9-like [*Strongylocentrotus purpuratus*] | KRAB Domain-containing zinc finger protein | DOWN | 2.05 | 0.22 |
|  | Unigene33252_All | Hypothetical protein BRAFLDRAFT_280890 [*Branchiostoma floridae*] | KRAB Domain-containing zinc finger protein | DOWN | 0.92 | 0.00 |
| C3H | CL9096.Contig2_All | Zinc finger CCCH domain-containing protein 15-like [*Strongylocentrotus purpuratus*] | Transcriptional regulator ATRX | UP | 3.56 | 14.81 |
|  | CL9096.Contig3_All | Zinc finger CCCH domain-containing protein 15-like [*Strongylocentrotus purpuratus*] | Transcriptional regulator ATRX | UP | 2.91 | 12.01 |
|  | CL4481.Contig4_All | Zinc finger CCCH domain-containing protein 10-like isoform 1 [*Strongylocentrotus purpuratus*] | Cleavage and polyadenylation specificity factor subunit 4 | UP | 2.04 | 8.17 |

Table S5. Continued.

Table S5. Continued next page

| Family | Unigene | NR description | KEGG description | Si_TT2 *vs.* Si_TT0 | Si_TT0 FPKM | Si_TT2 FPKM |
| --- | --- | --- | --- | --- | --- | --- |
|  | Unigene32202_All | UOS5/S1 protein, putative [*Perkinsus marinus* ATCC 50983] | Tristetraprolin | DOWN | 1.33 | 0.00 |
|  | Unigene25291_All | Hypothetical protein Y032_0118g746 [*Ancylostoma ceylanicum*] | Elastin | DOWN | 2.49 | 0.00 |
| CPP | Unigene21750_All | Hypothetical protein [*Paramecium tetraurelia* strain d4-2] | - | UP | 0.20 | 1.45 |
| FHA | Unigene34220_All | serine/threonine-protein kinase Chk2-like [*Strongylocentrotus purpuratus*] | Serine/threonine-protein kinase Chk2 | UP | 0.00 | 0.71 |
| LIM | Unigene33849_All | Hypothetical protein BRAFLDRAFT_57046 [*Branchiostoma floridae*] | KRAB Domain-containing zinc finger protein | UP | 0.18 | 1.14 |
|  | CL2120.Contig2_All | Lipoma-preferred partner-like isoform 2 [*Strongylocentrotus purpuratus*] | lipoma-preferred partner | UP | 0.21 | 1.17 |
|  | Unigene25534_All | Uncharacterized protein LOC100888350 [*Strongylocentrotus purpuratus*] | KRAB Domain-containing zinc finger protein | UP | 0.25 | 1.35 |
| MYB-related | CL7923.Contig2_All | Chromatin complexes subunit BAP18-like isoform 1 [*Strongylocentrotus purpuratus*] | - | UP | 0.26 | 1.86 |

Table S5. Continued.

Table S5. Continued on next page.

| Family | Unigene | NR description | KEGG description | Si_TT2 *vs.* Si_TT0 | Si_TT0 FPKM | Si_TT2 FPKM |
| --- | --- | --- | --- | --- | --- | --- |
| TIG | CL2247.Contig3_All | Extracellular domains-containing protein CG31004-like [*Strongylocentrotus purpuratus*] | Alpha-tectorin | UP | 1.91 | 22.88 |
|  | Unigene22403_All | Uncharacterized protein LOC590859 [*Strongylocentrotus purpuratus*] | Nuclear factor of activated T-cells 5 | DOWN | 11.17 | 1.98 |
| Trihelix | Unigene20297_All | Hypothetical protein BRAFLDRAFT_220166 [*Branchiostoma floridae*] | - | UP | 0.23 | 1.94 |

Table S5. Continued
